# Isolation, Identification and Preliminary Remediation Performance Evaluation of Heavy Metal-Tolerant Bacteria from the Mining Area in Northern Shaanxi

**DOI:** 10.3390/microorganisms14081741

**Published:** 2026-08-07

**Authors:** Yatan Qu, Haiyang Guo, Si Li, Na Wang, Xiaopeng Gao

**Affiliations:** School of Life Sciences, Yan’an University, Yan’an 716000, China; skyquyatan@163.com (Y.Q.); guoocean1999@163.com (H.G.); lisiyau@163.com (S.L.); yxmnnzs@outlook.com (N.W.)

**Keywords:** microbial remediation, heavy metal, extracellular adsorption, *Bacillus*

## Abstract

To address heavy metal contamination in soils of the northern Shaanxi coal mining area, this study directionally screened and isolated a functional bacterial strain, *Bacillus* sp. NM-1, tolerant to Mn^2+^, Fe^3+^, and Cr^6+^, from polluted soil collected in the Shenmu mining region, Shaanxi Province, China. The isolate was identified as *Bacillus* sp. based on 16S rRNA gene sequencing analysis. A subsequent pot experiment was conducted to systematically evaluate the remediation efficacy of the selected strain in manganese-contaminated soil. Among the six metal-tolerant strains obtained, strain NM-1 exhibited the highest removal efficiency and the broadest concentration adaptability for Mn^2+^, Fe^3+^, and Cr^6+^, achieving over 99.5% removal of Mn^2+^ at an initial concentration of 100 mg/L. Subcellular distribution analyses of intracellular and extracellular ions, together with inactivated cell controls, revealed that extracellular adsorption constituted the primary mechanism underlying heavy metal removal. In the pot experiment, manganese ion (Mn^2+^) was used as the model contaminant. The results demonstrated that inoculation with NM-1 significantly alleviated manganese-induced growth inhibition in maize plants, as evidenced by increased plant height, biomass, and photosynthetic pigment content. Furthermore, NM-1 inoculation reduced malondialdehyde (MDA) accumulation while enhancing peroxidase (POD) activity and proline content, thereby improving the plant’s antioxidant capacity and osmotic regulation. Overall, this study provides a multifunctional metal-tolerant bacterial strain that represents a valuable microbial resource and offers theoretical support for microbe–plant combined remediation strategies in heavy metal-contaminated soils of the northern Shaanxi mining region.

## 1. Introduction

As a core national energy and chemical industrial base in China, the northern Shaanxi region has long endured severe ecological pressure from large-scale coal mining, petroleum extraction, and high-intensity industrial operations, which has triggered a progressively severe heavy metal contamination crisis in local soil and aquatic environments [1]. The discharge of waste and wastewater during coal mining has caused pollution to the soil and environment, resulting in reduced soil fertility and hindered plant growth [2]. Furthermore, heavy metals in pollutants can accumulate in the human body through the food chain, which causes serious harm to human health [3,4,5]. Extensive research on heavy metal pollution remediation has been documented in the domestic and international academic studies [6,7]. As a critical branch in the field of environmental engineering, physicochemical remediation technologies for soil heavy metal contamination have attracted considerable scholarly attention due to their distinct advantages, such as high remediation efficiency, controllable operational parameters, and applicability to sites contaminated with high concentrations of heavy metals. However, these physicochemical methods inherently exhibit certain limitations and thus cannot achieve the fundamental elimination of heavy metal pollution [8,9]; In contrast, phytoremediation technologies using hyperaccumulator plants [10,11] can temporarily alleviate soil heavy metal contamination, but this approach is constrained by a prolonged remediation period and bears the potential risk of inducing secondary pollution [12]. Microorganisms have received considerable scholarly attention in the field of heavy metal pollution remediation, attributed to their inherent merits of short growth cycles, low remediation costs, and the unique capability to form a synergistic remediation system with plants for mitigating heavy metal contamination [13]. Microorganisms primarily accomplish the effective remediation of heavy metal contaminants through multiple physiological and metabolic mechanisms, such as biosorption [14], bioaccumulation, bioprecipitation [15], and redox transformation [16]. Notably, a subset of microorganisms exhibits dual functionalities, encompassing heavy metal tolerance and plant growth-promoting (PGP) capabilities [17,18]. These functional microorganisms can be further formulated into microbial inoculants by combining them with fertilizers and other auxiliary substrates [19,20], thereby facilitating their practical application in heavy metal-contaminated soil remediation.

As a typical representative of coal mining areas in northern Shaanxi, the Shenmu coal mining area is characterized by severe soil-combined heavy metal contamination [21]. At present, the research on heavy metal remediation mainly focuses on physical–chemical remediation technologies and single-species plant remediation strategies [22,23]. Notably, there remains a significant paucity of systematic studies focusing on the screening, identification, and underlying remediation mechanisms of multifunctional microbial strains with tolerance to Mn^2+^, Fe^3+^, and Cr^6+^. This research gap has consequently led to a scarcity of high-quality functional microbial strain resources that are directly applicable to the remediation of contaminated soil in this ecologically sensitive mining region. The introduction of exogenous bacteria poses a significant risk of disrupting the structure and functional diversity of native soil microbial communities, which represents a critical ecological constraint on its application potential [24,25,26]. Nevertheless, these ecological considerations have been largely overlooked in earlier studies. This study focused on heavy metal-contaminated soil collected from the Shenmu coal mining area in Shaanxi Province, with the aim of directionally screening microbial strains tolerant to Mn^2+^, Fe^3+^, and Cr^6+^. The isolated strains were characterized by 16S rRNA gene sequencing, and their capacities for heavy metal removal were systematically assessed. Furthermore, pot culture experiments were carried out to verify the regulatory effects of the screened strains on the growth performance and physiological characteristics of maize cultivated in Mn-contaminated soil. This study is anticipated to provide high-quality functional microbial resources and a theoretical basis for improving crop resilience in heavy metal-contaminated soils in coal mining areas of northern Shaanxi, thereby laying a foundation for the practical application of microbial agents in regional agricultural and ecological settings.

## 2. Materials and Methods

### 2.1. Materials

Soil samples were collected from the Shenmu coal mining area in Yulin City, Shaanxi Province, using a random sampling strategy. Nine sampling points were randomly selected, and soil was excavated to a depth of 20 cm at each point. After the removal of visible stones and plant roots, the samples were placed into sterile sealed bags, transported to the laboratory in a refrigerated incubator at 4 °C, and subsequently stored at 4 °C until further use. Soil organic matter (SOM) content was measured using the potassium dichromate oxidation method with external heating (NY/T 1121.6-2021) [27]. The oven-drying method was employed to determine soil water content [28]. Soil pH and electrical conductivity (EC) were determined using a pH meter (P901, YOKE INSTRUMENT, Shanghai, China) and a conductivity meter (DDB-303A, INSEA, Shanghai China), in accordance with NY/T 1121.2-2006 [29] and HJ 802-2016 [30], respectively. The concentration of manganese ions (Mn^2+^) was measured via the ammonium persulfate spectrophotometric method (GB 5749–2006) [31]. Comprehensive data on soil physicochemical properties are provided in Supplementary Table S1. All chemicals used in the experiment were of analytical reagent (AR) grade; the soil used for pot culture experiments was collected from the back mountain of Yan’an University in Baota District, Yan’an City, Shaanxi Province.

### 2.2. Methods

#### 2.2.1. Screening of Strains Tolerant to Heavy Metals

Ten grams of soil sample was placed into a conical flask containing 100 mL of LB liquid medium (Tryptone 10 g/L, NaCl 5 g/L, yeast extract 10 g/L, distilled water 1 L, pH 7.2). The mixture was incubated at 37 °C with shaking at 180 rpm for 24 h. Subsequently, 1 mL of the enriched culture solution was serially diluted with 9 mL of sterile water to obtain bacterial suspensions with dilution gradients of 10^−4^, 10^−5^ and 10^−6^. Then, 100 μL of each diluted suspension was spread onto LB solid medium plates, followed by incubation at a constant temperature of 37 °C. After cultivation, typical colonies were selected and purified by streak plate method to obtain pure single colonies. The isolated single colonies were inoculated into LB medium and cultured for 24 h. For all subsequent experiments, bacterial suspensions were cultivated to an optical density at 600 nm (OD_600_) of 0.8–1.0 prior to use. Subsequently, 100 μL of the 10^−6^ bacterial suspension was spread onto LB plates containing Mn^2+^ at concentrations of 0, 20, 40, 60, 80, and 100 mg/L, as well as Cr^6+^ and Fe^3+^ at concentrations of 0, 2, 4, 6, 8, and 10 mg/L, respectively. After incubation at 37 °C and 180 rpm for 24 h, the strains were isolated, purified, and numbered [32]. The manganese-tolerant strains were inoculated onto LB medium supplemented with Fe^3+^ and Cr^6+^, the iron-tolerant strains onto LB medium containing Mn^2+^ and Cr^6+^, and the chromium-tolerant strains onto LB medium amended with Mn^2+^ and Fe^3+^. The growth performance of all tested strains was subsequently observed and recorded.

#### 2.2.2. Removal Rate of Heavy Metal Ions by Strains

Ferric ion (Fe^3+^) was determined via the o-phenanthroline spectrophotometric method [33,34]; Manganese ion (Mn^2+^) was detected by the ammonium persulfate spectrophotometric method (GB 5749–2006); Hexavalent chromium (Cr^6+^) was measured using the diphenylcarbazide spectrophotometric method (GB 5749–2006). LB media containing Mn^2+^ were prepared at concentrations of 0, 20, 40, 60, 80, and 100 mg/L, as well as Cr^6+^ and Fe^3+^ at concentrations of 0, 2, 4, 6, 8, and 10 mg/L, respectively. Subsequently, 100 μL of NM-1 bacterial suspension was inoculated into the prepared media and incubated at 37 °C with shaking at 180 r/m for 24 h. Afterwards, 4.5 mL of the culture broth was collected from each flask and centrifuged at 8000 r/m for 20 min, and the concentrations of heavy metal ions in the resulting supernatant were determined.

#### 2.2.3. 16S rRNA Molecular Identification and Phylogenetic Tree Construction

Genomic DNA of the isolated strain NM-1 was extracted using a bacterial genomic DNA extraction kit (TIANGEN BIOTECH (Beijing) Co., Ltd., Beijing, China) in accordance with the manufacturer’s protocols. The 16S rRNA gene was amplified by polymerase chain reaction (PCR) with the universal primer pair 27F and 1492R. Each PCR was performed in a total volume of 20 μL reaction system containing 1 μL of genomic DNA template. The PCR thermal profile was set as follows: initial denaturation at 95 °C for 30 s, followed by 35 cycles of denaturation at 95 °C for 30 s, annealing at 55 °C for 30 s, and extension at 72 °C for 1 min. A final extension was conducted at 72 °C for 10 min. All PCR amplifications were implemented on a TC-96/G/H(b)C thermal cycler (BIOER, Hangzhou, China). Subsequently, the purified 16S rRNA amplification product of the strain NM-1 was sent to the company (Tsingke Biotechnology Co., Ltd., Beijing, China) for sequencing analysis. The obtained 16S rRNA gene sequence was subjected to homology alignment using the BLAST (https://blast.ncbi.nlm.nih.gov/Blast.cgi) program [35,36]. Phylogenetic relationships among 11 taxa were inferred using the Neighbor-Joining (NJ) method, and the optimal phylogenetic tree was constructed accordingly. Bootstrap values, calculated from 1000 replicate analyses, were marked at the corresponding branch nodes to indicate the support rate of each cluster. The phylogenetic tree was drawn to scale, with branch lengths consistent with the units of evolutionary distance used for tree construction. Evolutionary distances were computed using the p-distance method, with the units being the number of base differences per site. The analysis covered 11 nucleotide sequences, including the 1st, 2nd and 3rd codon positions, as well as non-coding sites. The partial deletion method was applied to eliminate sites with coverage lower than 50%, and the final aligned dataset contained 1338 valid sites. All evolutionary analyses were performed in MEGA12 software [37].

#### 2.2.4. Quantitative Assay of Extracellular and Intracellular Manganese Ions

Strain NM-1 was inoculated into LB liquid medium supplemented with Mn^2+^ at a final concentration of 100 mg/L, and cultured with shaking at 180 r/min and 37 °C for 24 h. Upon completion of incubation, the bacterial culture was centrifuged at 10,000 r/min for 5 min to harvest bacterial cells. The collected bacterial cells were repeatedly washed with 0.1 M HCl to completely remove Mn^2+^ adsorbed on the cell surface. Subsequently, the bacterial cells were rinsed with 10% SDS to eliminate residual acid on the cell surface, and then resuspended in 10% SDS solution. The bacterial cells were subjected to repeated freeze–thaw cycles at −20 °C and 37 °C to achieve complete cell lysis. After cell disruption, the mixture was centrifuged at 10,000 r/min for 5 min, and the supernatant was carefully collected. The concentrations of Mn^2+^ in the washing solution and the collected supernatant were determined respectively by the previously described spectrophotometric method. Another aliquot of the culture in the logarithmic growth phase was harvested by centrifugation at 10,000 r/min for 5 min. The collected cells were washed two to three times with sterile PBS and then resuspended to the desired concentration (10^8^ CFU/mL). The bacterial suspension was sterilized at 121 °C for 21 min; 100 μL of the suspension was spread onto LB medium to verify the absence of viable colonies. If no colony growth was observed, both live and inactivated bacteria prepared at the same stage were inoculated separately into LB medium containing 100 mg/L Mn^2+^ and incubated at 37 °C with shaking at 180 r/min for 24 h. After incubation, the Mn^2+^ removal efficiencies of the live and inactivated bacteria were determined.

#### 2.2.5. Pot Experiment Design

Soil, perlite, and sand were thoroughly mixed to ensure homogeneity. Each pot was filled with 800 g of the prepared soil mixture and supplemented with 200 g of chicken manure fertilizer. Maize seeds (Zhongnong Tian 488) were surface-sterilized by soaking in 10% (v/v) H_2_O_2_ for 10 min, followed by rinsing with sterile water. Six maize seeds were then sown in each pot according to the method described previously [38]. Ten days after seedling emergence, the plants were thinned to retain three uniformly growing seedlings per pot. Maize plants with similar growth status were selected and treated according to the conditions listed in Table 1. The pot experiment was conducted for a total of 30 days.

#### 2.2.6. Data Analysis and Graphic Production

Statistical analyses were performed using IBM SPSS Statistics 27. One-way analysis of variance (ANOVA) was applied to evaluate differences among treatments, and mean comparisons were conducted using Duncan’s multiple range test at a significance level of 0.05. Graphs were generated using OriginPro 2021.

## 3. Results

### 3.1. Isolation and Screening of Heavy Metal-Tolerant Strains

**Figure 1:**
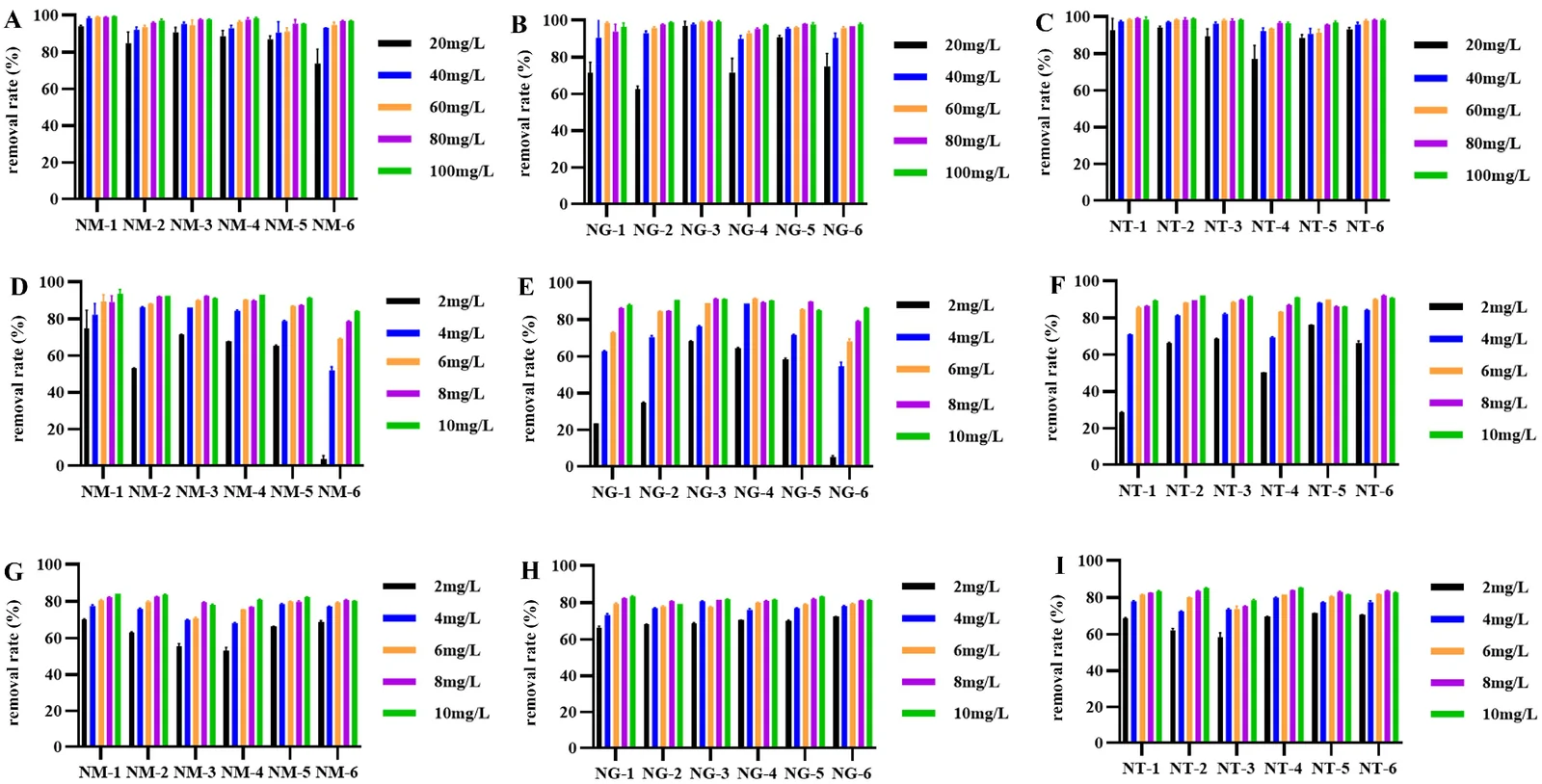
The removal rates of metal ions by different strains are shown in the figure. Panels (**A**–**C**) represent the removal rates of Mn^2+^, panels (**D**–**F**) represent the removal rates of Cr^6+^, and panels (**G**–**I**) represent the removal rates of Fe^3+^. Error bars represent standard deviation (SD, n = 3).

A total of six manganese-resistant strains (NM-1, NM-2, NM-3, NM-4, NM-5, NM-6), six iron-resistant strains (NT-1, NT-2, NT-3, NT-4, NT-5, NT-6), and six chromium-resistant strains (NG-1, NG-2, NG-3, NG-4, NG-5, NG-6) were obtained through isolation and screening (Figure 1). To evaluate the multi-metal tolerance of these strains, the manganese-resistant strains were inoculated on LB agar containing Fe^3+^ and Cr^6+^, the iron-resistant strains were inoculated on LB agar containing Mn^2+^ and Cr^6+^, and the chromium-resistant strains were inoculated on LB agar containing Mn^2+^ and Fe^3+^. All strains exhibited normal growth under incubation at 37 °C, indicating that the selected strains possess combined tolerance to Mn^2+^, Fe^3+^, and Cr^6+^.

### 3.2. Determination of Heavy Metal Ion Removal Capacity of Strains

The removal efficiencies of Mn^2+^, Cr^6+^, and Fe^3+^ by 18 tolerant strains at different initial concentrations (Figure 1) indicated that strain NM-1 exhibited the best overall performance. For Mn^2+^ removal (Figure 1A–C), the NM strains significantly outperformed the NG and NT series. Notably, NM-1 maintained removal rates above 90% across the full concentration range of 20–100 mg/L, achieving over 99.5% at 100 mg/L, demonstrating both the highest stability and efficiency. For Cr^6+^ removal (Figure 1D–F), NM-1 consistently outperformed other strains across the 2–10 mg/L concentration range, reaching nearly 95% removal at 10 mg/L. While the removal efficiencies of other strains generally increased with concentration, they remained lower than NM-1, and some strains (e.g., NM-6) exhibited removal rates below 10% at low concentrations, indicating limited tolerance. In the case of Fe^3+^ removal (Figure 1G–I), all strains showed increasing removal rates with rising concentrations, with overall differences being relatively small. NM-1 consistently achieved the highest efficiency across all concentrations, exceeding 80% at 10 mg/L, whereas other series generally ranged between 60% and 80%, showing less sensitivity to concentration. Collectively, NM-1 demonstrated the highest removal efficiency and broad concentration adaptability for Mn^2+^, Cr^6+^, and Fe^3+^, highlighting its potential for remediation of multi-metal contaminated environments.

### 3.3. Results of 16S rRNA Molecular Identification

**Figure 2:**
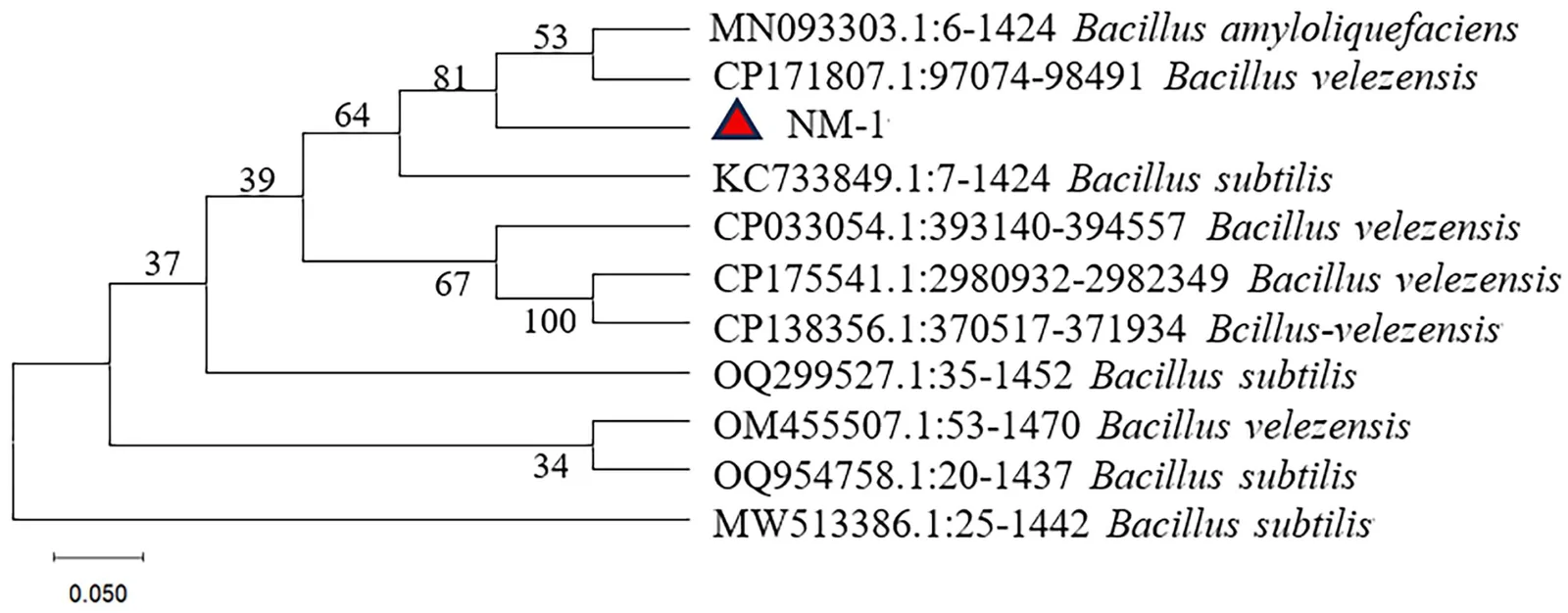
The red triangle in the figure indicates the target strain NM-1, and the remaining strains are reference strains obtained from the GenBank database. Bootstrap support values (based on 1000 replicates) are shown at the branch nodes. The scale bar at the bottom represents genetic distance, with a unit length corresponding to 0.05 nucleotide substitutions per site. Longer branch lengths indicate greater sequence divergence and larger evolutionary distances.

A phylogenetic tree was constructed based on 16S rRNA gene sequences (Figure 2), using reference *Bacillus* strains from the NCBI database. The isolated strain NM-1 was classified and identified through this analysis. The results showed that NM-1 clustered within the same branch as *Bacillus velezensis* (reference sequence CP171807.1:97074–98491), with a bootstrap value of 81, indicating high sequence homology. Based on the comprehensive phylogenetic analysis, strain NM-1 was identified as *Bacillus* sp.

### 3.4. Quantitative Determination of Extracellular and Intracellular Manganese Ions

**Figure 3:**
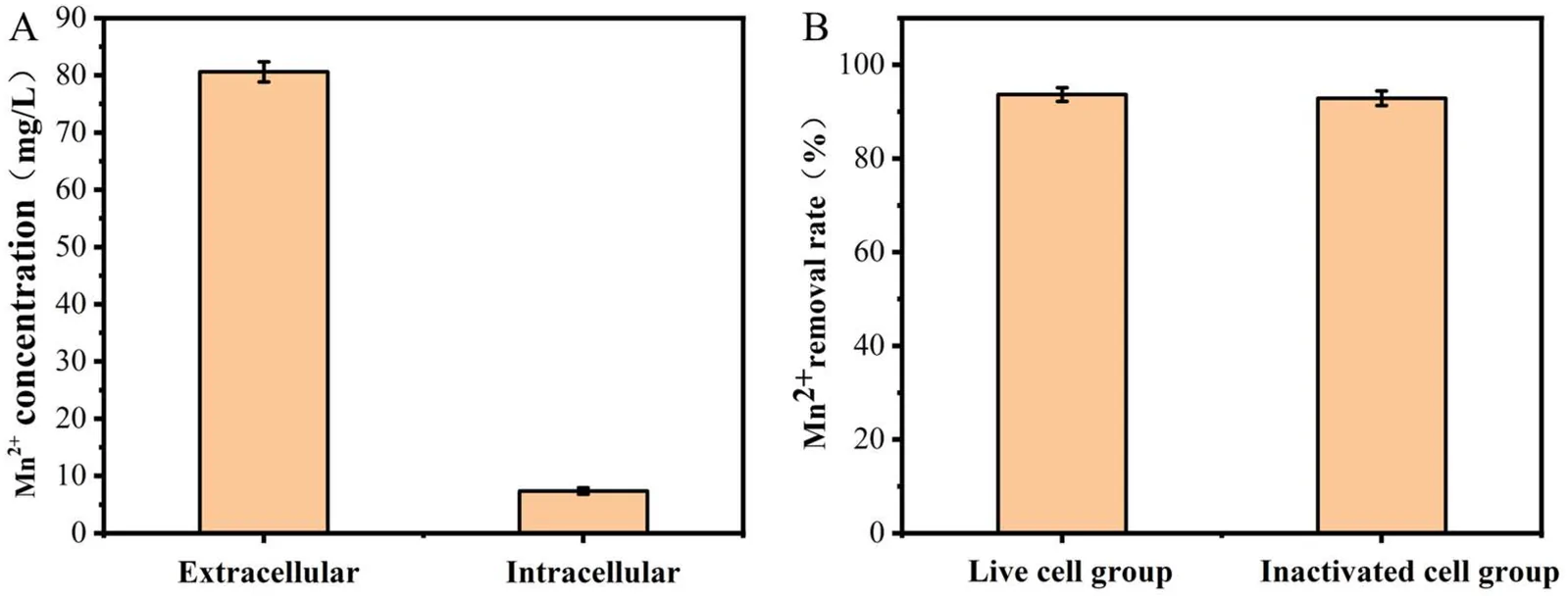
(**A**) Intracellular and extracellular manganese ion concentrations; (**B**) Removal rates of manganese ions from liquid medium by live cells and heat-inactivated cells of strain NM-1. Error bars represent standard deviation (SD, n = 3).

The extracellular and intracellular Mn^2+^ contents were quantified as 80.60 mg/L and 7.37 mg/L (Figure 3A), respectively, with extracellular concentrations far exceeding intracellular levels. This significant difference indicates that NM-1 primarily removes Mn^2+^ through extracellular adsorption. To further verify this mechanism, the removal efficiencies of live and inactivated cells were measured at 100 mg/L Mn^2+^, yielding 93.65% and 92.88%, respectively (Figure 3B). The comparable removal rates suggest that Mn^2+^ removal by NM-1 is predominantly mediated via extracellular adsorption and is largely independent of metabolic activity or intracellular accumulation.

### 3.5. Pot Experiment

**Figure 4:**
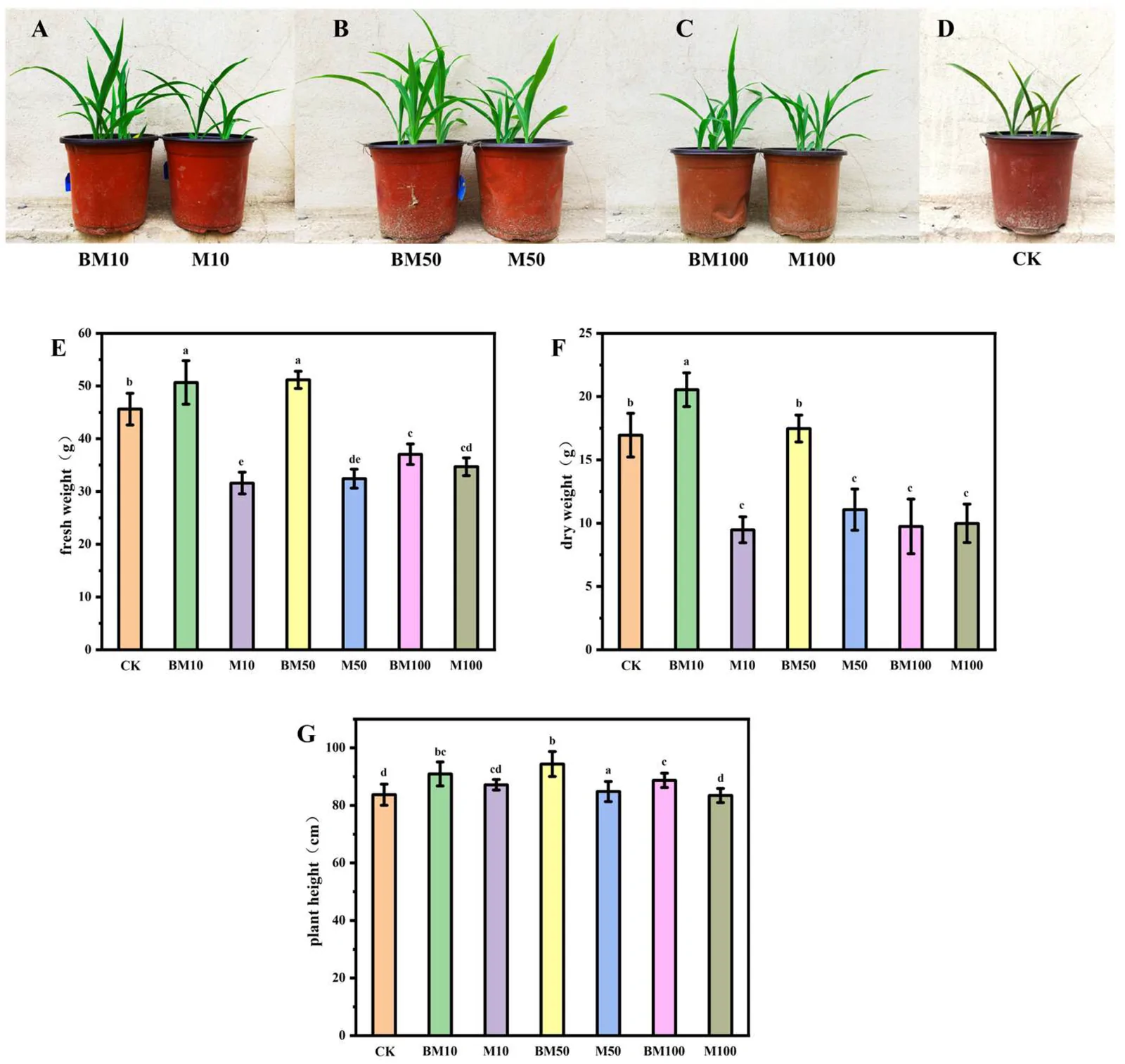
Phenotypes of maize plants under different treatments after 30 days of growth (**A**–**D**). Fresh weight (**E**), dry weight (**F**), and shoot height (**G**) of maize plants under different treatments. Lowercase letters indicate significant differences at the *p* < 0.05 level. Error bars represent standard deviation (SD, n = 6).

As can be seen from Figure 4A–C, the corn plants in the groups treated with the bacterial strain (BM10, BM50, BM100) exhibited significantly greater height than those in the untreated groups (M10, M50, M100). Biomass analysis further indicated that the fresh and dry weights of maize in the non-inoculated groups (M10, M50, M100) were consistently lower than those in the inoculated groups (BM10, BM50, BM100). Inoculation with NM-1 significantly increased biomass across all treatment groups. In the BM10 group, fresh weight (50.6 g) and dry weight (20.5 g) increased by 59.1% and 118.09%, respectively, compared with M10. In the BM50 group, fresh weight (51.2 g) and dry weight (17.4 g) were 57.72% and 56.76% higher than M50. In the BM100 group, fresh weight (37.1 g) and dry weight (9.7 g) increased by 6.92% and −2.06% relative to M100 (Figure 4E,F). Plant height measurements indicated that Mn^2+^ stress significantly inhibited maize growth; heights in BM10 (90.9 cm), BM50 (94.4 cm), and BM100 (88.7 cm) were increased by 4.24%, 11.32%, and 6.35%, respectively, compared with the corresponding non-inoculated groups (Figure 4G).

**Figure 5:**
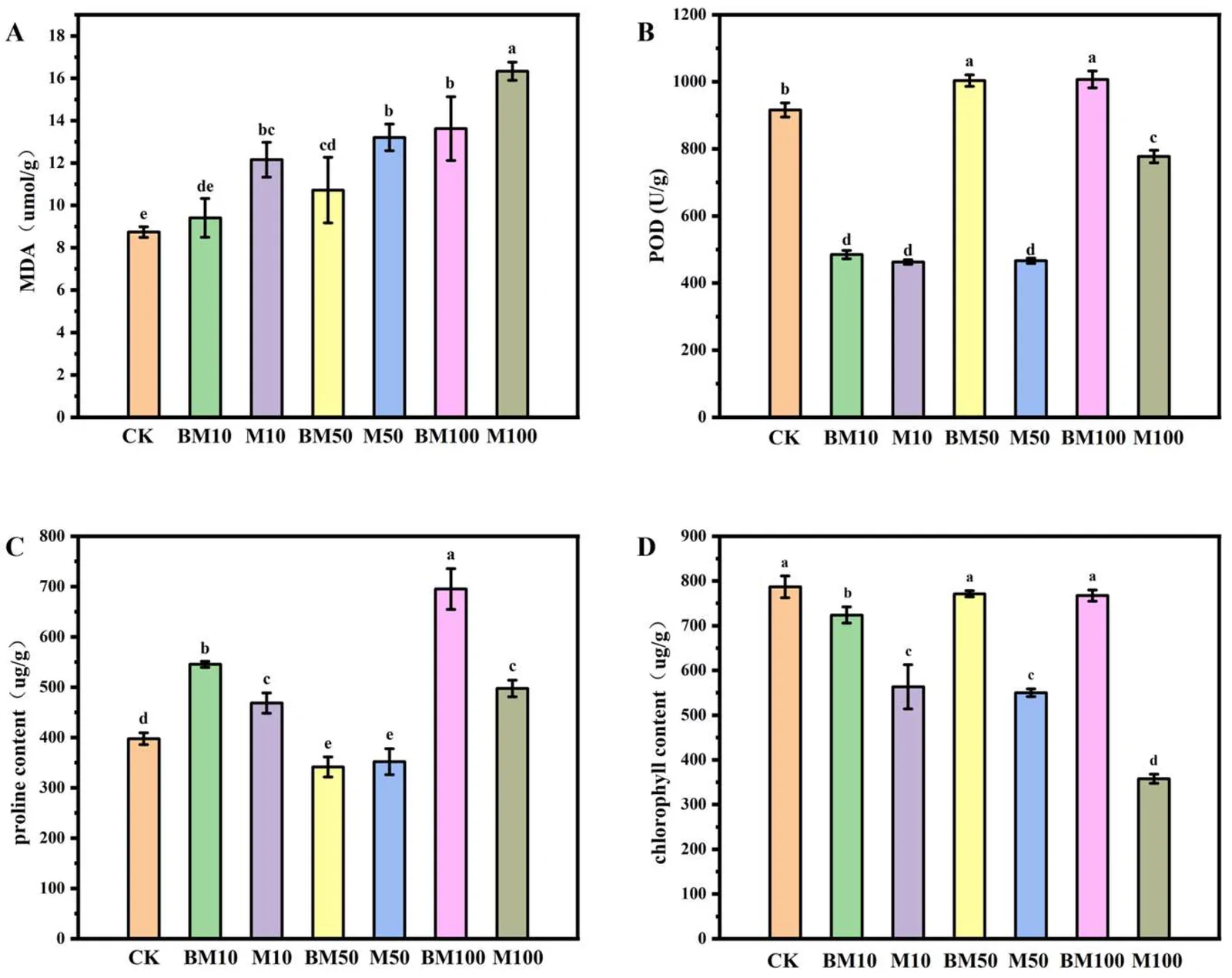
Physiological indices of maize plants. (**A**) MDA content; (**B**) POD activity; (**C**) proline content; (**D**) chlorophyll content. Lowercase letters indicate significant differences at the *p* < 0.05 level. Error bars represent standard deviation (SD, n = 3).

Manganese stress impaired physiological metabolism including antioxidant defense, osmotic regulation and photosynthetic pigment synthesis in maize, while NM-1 inoculation relieved manganese-induced oxidative damage and optimized plant physiological conditions (Figure 5A). Relative to the control, MDA content increased significantly in a concentration-dependent manner under manganese stress, reflecting severe cell membrane injury caused by excess manganese. Under equivalent manganese treatments, plants inoculated with NM-1 exhibited distinctly lower MDA concentrations than uninoculated counterparts, with values of 9.4 μmol/g (BM10), 10.7 μmol/g (BM50), and 13.6 μmol/g (BM100), respectively, compared to 12.2 μmol/g (M10), 13.2 μmol/g (M50), and 16.3 μmol/g (M100). These results confirm that strain NM-1 can efficiently relieve manganese-triggered lipid peroxidation. Figure 5B illustrates the impact of manganese stress on antioxidant enzyme peroxidase (POD) activity in maize. Exposure to manganese stress significantly altered the POD response. Specifically, POD activities in the M10 and M50 treatments (458–469 U/g) were markedly lower than that in the control group (916 U/g). However, inoculation with strain NM-1 led to a significant increase in POD activity under all manganese treatments. Notably, POD activities in the BM50 (1003 U/g) and BM100 (1007 U/g) treatments recovered to levels close to the control, while the BM10 treatment (485 U/g) exhibited only a slight increase relative to the M10 treatment. The proline content (Figure 5C) in maize plants under the BM100 treatment (with bacterial inoculation) was 695.26 µg/g, which was 39.76% higher than that of the M100 group (497.45 µg/g). Manganese stress caused a pronounced decline in maize chlorophyll content. All manganese treatments exhibited notably lower chlorophyll concentrations than the control (787 μg/g); M100 showed the most severe decrease. NM-1 inoculation effectively restored chlorophyll accumulation under manganese stress. Relative to uninoculated counterparts, chlorophyll contents in BM10, BM50 and BM100 were elevated by 28.47%, 40.22% and 114.5%, reaching 723 μg/g, 771 μg/g and 767 μg/g in sequence (Figure 5D). Inoculation with NM-1 mitigates oxidative damage and photosynthetic inhibition caused by manganese stress in maize, and improves plant tolerance to heavy metals. The underlying mechanisms include reduced MDA content, enhanced POD activity and proline levels, as well as maintained chlorophyll content. This suggests that NM-1 helps maintain or restore plant antioxidant enzyme activity, thereby strengthening reactive oxygen species (ROS) scavenging capacity. Furthermore, proline, as a key osmolyte, exhibits dynamic changes in content that reflect the plant’s adaptive response to stressful environments.

## 4. Discussion

In this study, heavy metal-contaminated soil from the Shenmu mining area in northern Shaanxi was used to directionally screen 18 functional bacterial strains tolerant to Mn^2+^, Fe^3+^, or Cr^6+^. All strains exhibited combined tolerance to the three heavy metals, which is consistent with the findings of Na Wang et al. [1] regarding multi-heavy metal co-contamination in the northern Shenmu mining area. This result also confirms the presence of abundant heavy metal-tolerant microbial resources in the contaminated soils of the mining area, providing a foundational strain repository for the future development of microbial remediation technologies. Among the isolates, strain NM-1 demonstrated the highest heavy metal removal efficiency and concentration adaptability, achieving a removal rate of over 99.5% under 100 mg/L Mn^2+^ stress, highlighting its potential advantage in the remediation of Mn^2+^ contamination. The initial pH value is known to exert a considerable influence on the removal efficiency of heavy metal ions [8]. Although this factor was overlooked in our preliminary manganese removal experiments, its influence will be systematically examined in follow-up studies.

Strain identification revealed that NM-1 belongs to *Bacillus* sp. The genus *Bacillus* encompasses a group of closely related species, including *Bacillus subtilis, B. amyloliquefaciens*, *B. licheniformis*, *B. velezensis*, *and B. atrophaeus*, among others. The 16S rRNA gene sequences among these species exhibit extremely high similarity, frequently exceeding 99% [39,40]. Given the extremely low interspecific divergence, accurate identification of NM-1 at the species level cannot be achieved based solely on 16S rRNA gene sequencing. To resolve this taxonomic ambiguity, whole-genome sequencing will be performed in our subsequent work to further clarify the phylogenetic affiliation of strain NM-1. Strains of the genus *Bacillus* are widely used in the remediation of heavy metal-contaminated soils due to their strong stress tolerance, rapid reproduction, and ability to produce various metabolites [41,42]. This study confirmed that NM-1 removes heavy metals primarily through extracellular adsorption, with less dependence on intracellular accumulation or active metabolic processes. Quantification of manganese ions inside and outside the cells showed that the extracellular manganese concentration (80.60 mg/L) was significantly higher than the intracellular concentration (7.37 mg/L). Furthermore, no significant difference in heavy metal removal efficiency was observed between live and inactivated cells (93.65% vs. 92.88%). These results suggest that the removal of heavy metals by NM-1 occurs primarily via extracellular adsorption, with limited apparent involvement of intracellular metabolic pathways. This finding is in agreement with previous observations [43] that extracellular adsorption serves as a key non-metabolic route in microbial heavy metal remediation. This mechanism appears to be largely independent of cellular metabolic activity and is more likely attributable to the physicochemical properties of the cell surface. The cell walls of *Bacillus* species are known to harbor a variety of functional groups [44], which have been suggested as valuable moieties in environmental remediation and biotechnological contexts. For instance, Bai Jun et al. [45] demonstrated that carboxyl, carbonyl, hydroxyl, amino, and phosphate groups on the surface of *Bacillus subtilis* strain DBM are involved in the immobilization of soil Cu^2+^. Based on these findings, we hypothesize that NM-1 may bind Mn^2+^ through functional groups such as hydroxyl and carboxyl groups on its cell wall surface [46], thereby adsorbing and immobilizing the heavy metal ion and subsequently alleviating its toxicity to plants. The specific adsorption sites and underlying mechanisms require further investigation.

The pot experiment confirmed that inoculation with NM-1 effectively alleviated the inhibitory effects of Mn^2+^ stress on maize growth, significantly increasing plant height, biomass, and photosynthetic pigment content. This finding is consistent with the report by Anna Muratova et al. [47] that microbial agents can improve plant growth in heavy metal-contaminated soils. Under low-to-medium Mn^2+^ contamination (10 and 50 mg·kg^−1^), the fresh and dry weights of maize increased by more than 56% following NM-1 inoculation, indicating a particularly pronounced remediation effect. However, under high-level contamination (100 mg·kg^−1^), the increase in dry weight was not significant. Ying Liu et al. [48] demonstrated that manganese toxicity significantly enhances the accumulation of manganese and iron in plant roots. It is therefore hypothesized that high concentrations of Mn^2+^ may cause irreversible damage to maize roots [49], making it difficult to fully reverse the toxic effects even with NM-1 inoculation. These results suggest that NM-1 is more suitable for the remediation of soils contaminated with low-to-medium concentrations of Mn^2+^. For application to highly contaminated sites, co-application with a soil conditioner may be considered to further enhance remediation efficiency [50,51].

Under manganese stress, plants generate excessive reactive oxygen species, triggering lipid peroxidation and leading to elevated malondialdehyde (MDA) content [52], while simultaneously disrupting the photosynthetic system and inhibiting antioxidant enzyme activity. This study demonstrates that inoculation with NM-1 effectively alleviates these damages by regulating plant physiological metabolism. Specifically, following NM-1 inoculation, MDA content decreased significantly in all treatment groups, while POD activity and proline content increased markedly, and chlorophyll content was enhanced by more than 164% compared with the non-inoculated groups. These results indicate that NM-1 alleviates Mn^2+^-induced oxidative damage by enhancing both antioxidant capacity and osmotic adjustment, thereby maintaining normal photosynthesis and growth metabolism. Notably, the chlorophyll content in the BM50 treatment group was significantly higher than that in the control (CK), further suggesting that NM-1 may alleviate heavy metal stress and improve soil micro-environmental conditions, thereby facilitating nutrient acquisition and supporting plant physiological performance. This finding is consistent with the report by Saikat Mazumder et al. [53] on the synergistic effects of microbe-plant interactions in improving soil fertility and plant stress tolerance. Furthermore, under high-concentration Mn^2+^ stress (M100 group), POD activity in maize showed a rebound, which is speculated to be a stress-induced defense response to heavy metal toxicity [54]. Previous studies have demonstrated that excessive manganese concentrations trigger cellular defense mechanisms, upregulate the expression of antioxidant enzyme genes, and consequently enhance the activity of corresponding antioxidant enzymes to scavenge excess reactive oxygen species [48]. This mechanism is consistent with the observed increase in POD activity under high Mn^2+^ concentration in the present experiment. After inoculation with NM-1, POD activity in the BM100 group further increased to 1007 U/g, approaching the control (CK) level, indicating that NM-1 enhances the plant’s stress defense capacity and reduces heavy metal-induced cellular damage.

The innovation of this study lies in the directional screening of *Bacillus* sp. strain NM-1 with combined tolerance to Mn^2+^, Fe^3+^, and Cr^6+^ from the Shenmu mining area in northern Shaanxi, the elucidation of its extracellular adsorption mechanism, and the verification of its application potential in microbe-plant combined remediation through pot experiments. These findings provide high-quality microbial resources and theoretical support for the remediation of multi-heavy metal-contaminated soils in the Shenmu mining area. However, several limitations remain. First, strain screening was confined to a single area of the Shenmu mining site, and the narrow sampling range may limit the representativeness of the isolated strain. Second, only the remediation efficacy of NM-1 on Mn^2+^-contaminated soil was examined, while its performance under Fe^3+^, Cr^6+^, or combined pollution remains unknown. Third, the specific mechanism of extracellular adsorption has not been thoroughly investigated, and the types and functions of the adsorption-related functional groups remain unidentified. Fourth, the pot experiment period was short (30 days), and the long-term remediation stability of the strain and its effects on soil microbial community structure require further study.

Future research should extend pot-scale experiments to field trials to validate the long-term remediation performance and environmental adaptability of NM-1 under realistic conditions. In parallel, the development of composite remediation agents through the combination of NM-1 with biochar, organic fertilizer, or other synergistic materials warrants further investigation to improve overall remediation efficiency. Equally important are the practical challenges associated with large-scale field application, including formulation stability, delivery methods, and economic feasibility, which must be systematically addressed to enable viable and sustainable remediation strategies for heavy metal-contaminated soils in the Shenmu mining area.

## 5. Conclusions

In this study, six bacterial strains exhibiting tolerance to Mn^2+^, Fe^3+^, and Cr^6+^ were isolated from heavy metal-contaminated soil collected from the Shenmu mining area in northern Shaanxi. All isolates showed varying degrees of cross-tolerance to multiple heavy metals under the conditions tested. Strain NM-1, identified as a *Bacillus* sp., exhibited relatively high removal efficiency for Mn^2+^, reaching over 99.5% at a spiked concentration of 100 mg/L, although this value should be interpreted with caution given the controlled laboratory setting. Preliminary characterization of the underlying process pointed toward extracellular adsorption as a potentially dominant pathway, whereas the involvement of intracellular uptake appeared to be limited based on the current dataset. Physiological assays revealed that manganese stress significantly inhibited maize growth and disrupted the plant’s antioxidant defense system. However, inoculation with strain NM-1 markedly increased plant height and biomass, reduced malondialdehyde (MDA) content, and elevated peroxidase (POD) activity and proline accumulation. These results suggest that NM-1 effectively alleviates manganese-induced oxidative damage and enhances plant stress tolerance, with the most pronounced effects observed under low to moderate manganese contamination. Overall, strain NM-1 represents a promising microbial resource and provides a theoretical foundation for microbe–plant combined bioremediation strategies in heavy metal-contaminated soils of the Shenmu mining area, demonstrating considerable potential for field application.

## Figures and Tables

**Table 1:** Group setting for pot experiments.

Group	Treatment	Pollution Level (mg·kg^−1^)
CK	ultrapure water	0
M10	200 mL 0.05 mg/mL MnSO_4_	MnSO_4_ (10)
BM10	200 mL 0.05 mg/mL MnSO_4_ + 200 mL Bacterial suspension ^1^	MnSO_4_ (10)
M50	200 mL 0.25 mg/mL MnSO_4_	MnSO_4_ (50)
BM50	200 mL 0.25 mg/mL MnSO_4_ + 200 mL Bacterial suspension ^1^	MnSO_4_ (50)
M100	200 mL 0.50 mg/mL MnSO_4_	MnSO_4_ (100)
BM100	200 mL 0.50 mg/mL MnSO_4_ + 200 mL Bacterial suspension ^1^	MnSO_4_ (100)

^1^ Strain NM-1 was inoculated into the culture medium and incubated for 48 h. The bacterial cells were then harvested by centrifugation, the supernatant was discarded, and the pellet was resuspended in sterile physiological saline.

## Data Availability

The original contributions presented in this study are included in the article/Supplementary Material. Further inquiries can be directed to the corresponding author.

## Supplementary Materials

The following supporting information can be downloaded at: https://www.mdpi.com/article/10.3390/microorganisms14081741/s1, Figure S1: Picture of a single colony of the strain. NM1-6 is a strain that is tolerant to manganese ions; NT1-6 is an iron-ion-tolerant strain; NG1-6 is a strain tolerant to chromium ions. Figure S2: Physiological and biochemical characteristics of strain NM-1. A: VP test (positive); B: methyl red test (negative); C: indole test (positive); D: glucose fermentation (positive); E: sucrose fermentation (positive). Figure S3: Gram staining result of strain NM-1 (positive, purple). Figure S4: The 16S rRNA gene sequence of strain NM-1. Figure S5: Standard curves for the determination of (A) chromium, (B) manganese, and (C) iron ions. Table S1: Soil physicochemical properties.
