# Supplementary material for: Isolation, Identification and Preliminary Remediation Performance Evaluation of Heavy Metal-Tolerant Bacteria from the Mining Area in Northern Shaanxi
Source: Microorganisms. 2026 Aug 7;14(8):1741. doi: 10.3390/microorganisms14081741 (PMC13515670; doi:10.3390/microorganisms14081741)
Supplement: Supplementary file 1 [file microorganisms-14-01741-s001.zip › microorganisms-4439289-supplementary.pdf]

Supplementary Materials

Isolation, Identification and Preliminary Remediation Performance Evaluation of Heavy Metal-Tolerant Bacteria from the Mining Area in Northern Shaanxi

Yatan Qu1┼, Haiyang Guo1┼, Si Li1, Na Wang1, Xiaopeng Gao1*

[1]School of Life Sciences, Yan'an University, Yan'an, Shaanxi, China

┼These authors contributed equally to this work

***Correspondence: xiaopenggao@yau.edu.cn**

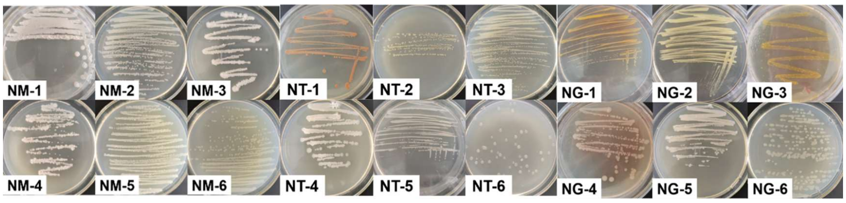

Supplementary Figure S1. Picture of a single colony of the strain. NM1-6 is a strain that is tolerant to manganese ions; NT1-6 is an iron-ion-tolerant strain; NG1-6 is a strain tolerant to chromium ions.

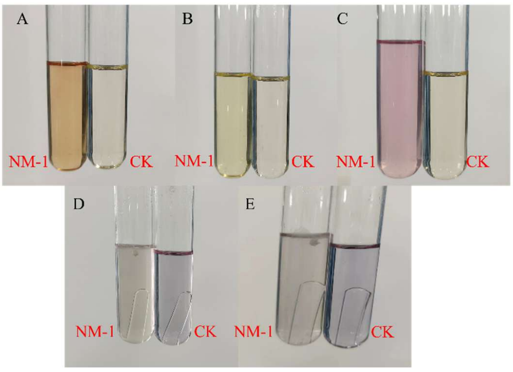

Supplementary Figure S2. Physiological and biochemical characteristics of strain NM-1. A: VP test (positive); B: methyl red test (negative); C: indole test (positive); D: glucose fermentation (positive); E: sucrose fermentation (positive).

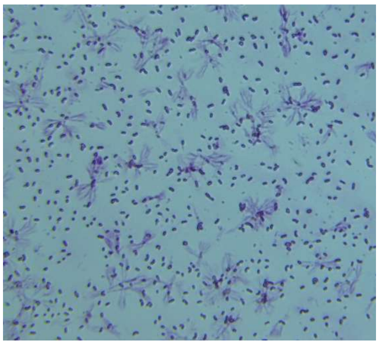

Supplementary Figure S3. Gram staining result of strain NM-1 (positive, purple).

```
>JUN7 TSS20230522-029-03855
CAGTCGAGCGGACAGATGGGAGCTTGCTCCCTGATGTTAGCGGCGGACGGGTGAGTAACACGTGGGTAACCTGCCTGTAAGACTGGGATAACTC
CGGGAAACCGGGGCTAATACCGGATGGTTGTTTGAACCGCATGGTTCAGACATAAAAGGTGGCTTCGGCTACCACTTACAGATGGACCCGCGGC
GCATTAGCTAGTTGGTGAGGTAACGGCTCACCAAGGCAACGATGCGTAGCCGACCTGAGAGGGTGATCGGCCACACTGGGACTGAGACACGGC
CCAGACTCCTACGGGAGGCAGCAGTAGGGAATCTTCCGCAATGGACGAAAGTCTGACGGAGCAACGCCGCGTGAGTGATGAAGGTTTTCGGATC
GTAAAGCTCTGTTGTTAGGGAAGAACAAGTGCCGTTCAAATAGGGCGGCACCTTGACGGTACCTAACCAGAAAGCCACGGCTAACTACGTGCCAG
CAGCCGCGGTAATACGTAGGTGGCAAGCGTTGTCCGGAATTATTGGGCGTAAAGGGCTCGCAGGCGGTTTCTTAAGTCTGATGTGAAAGCCCCC
GGCTCAACCGGGGAGGGTCATTGGAAACTGGGGAACTTGAGTGCAGAAGAGGAGAGTGGAATTCCACGTGTAGCGGTGAAATGCGTAGAGATG
TGGAGGAACACCAGTGGCGAAGGCGACTCTCTGGTCTGTAACTGACGCTGAGGAGCGAAAGCGTGGGGAGCGAACAGGATTAGATACCCTGGT
AGTCCACGCCGTAAACGATGAGTGCTAAGTGTTAGGGGGTTTCCGCCCCTTAGTGCTGCAGCTAACGCATTAAGCACTCCGCCTGGGGAGTACGG
TCGCAAGACTGAAACTCAAAGGAATTGACGGGGGCCCGCACAAGCGGTGGAGCATGTGGTTTAATTCGAAGCAACGCGAAGAACCTTACCAGGT
CTTGACATCCTCTGACAATCCTAGAGATAGGACGTCCCCTTCGGGGGCAGAGTGACAGGTGGTGCATGGTTGTCGTCAGCTCGTGTCGTGAGATG
TTGGGTTAAGTCCCGCAACGAGCGCAACCCTTGATCTTAGTTGCCAGCATTCAGTTGGGCACTCTAAGGTGACTGCCGGTGACAAACCGGAGGAA
GGTGGGGATGACGTCAAATCATCATGCCCCTTATGACCTGGGCTACACACGTGCTACAATGGGCAGAACAAAGGGCAGCGAAACCGCGAGGTTA
AGCCAATCCCACAAATCTGTTCTCAGTTCGGATCGCAGTCTGCAACTCGACTGCGTGAAGCTGGAATCGCTAGTAATCGCGGATCAGCATGCCGC
GGTGAATACGTTCCCGGGCCTTGTACACACCGCCCGTCACACCACGAGAGTTTGTAACACCCGAAGTCGGTGAGGTAACCTTTTAGGAGCCAGCC
GCCGAA
```

Supplementary Figure S4. The 16S rRNA gene sequence of strain NM-1.

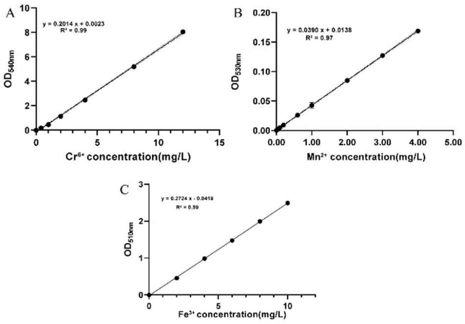

Supplementary Figure S5. Standard curves for the determination of (A) chromium, (B) manganese, and (C) iron ions.

Commented [M1]: We revised the second Figure S4 to Figure S5, please confirm.

Supplementary Table S1 soil physicochemical properties

Group	pH	EC（μS/cm）	Soil organic matter (g/kg)	Soil water content (%)	Mn^{2+} content（mg/kg）
Value[1]	7.97	22.86	7.99	6.65	115.33

[1]Each experiment was performed in triplicate, and the results were expressed as mean values.

Commented [M2]: Table in Supplementary Materials should be numbered starting from S1, we revised Table S4 to Table S1, please confirm.
